# Same-Sex Sexual Behavior in Invertebrates: A Systematic Synthesis of Prevalence, Study Type, and Interpretation

**DOI:** 10.3390/insects17060611

**Published:** 2026-06-10

**Authors:** Valeria Palma-Onetto, Constanza Millán-Medina, Shakil Ahmad, Viviana Rivera-Estay

**Affiliations:** 1 Departamento de Química y Medio Ambiente, Universidad Técnica Federico Santa María, Avenida España 1680, Valparaíso 2390123, Chile; 2 Programa de Doctorado en Ciencias con Mención en Biodiversidad y Biorrecursos, Facultad de Ciencias, Universidad Católica de la Santísima Concepción, Concepción 4070558, Chile; cmillan@ucsc.cl; 3 Guangdong Key Laboratory of Animal Conservation and Resource Utilization, Institute of Zoology, Guangdong Academy of Science, Guangzhou 510260, China; mshakil@aup.edu.pk; 4 Facultad de Ciencias, Universidad Arturo Prat, Iquique 1110939, Chile; vriverae@unap.cl

**Keywords:** invertebrates, sexual behavior, same-sex sexual behavior, behavioral ecology, mating systems

## Abstract

Same-sex sexual behavior in invertebrates includes interactions such as courtship, mounting, copulation, tandem running, and pair formation between individuals of the same sex. We conducted a systematic synthesis of 222 primary studies and 12 reviews and found published evidence of SSB in approximately 207 species spanning arthropods, mollusks, annelids, echinoderms, nematodes, and flatworms. Although most reports involved insects (approximately 89%), standardized reporting rates suggest this bias reflects research effort rather than true biological prevalence. Observations came from field studies, although several taxa, particularly Diptera and Hymenoptera, were disproportionately represented by laboratory work. Copulation-like interactions were the most frequently reported behavior, followed by courtship and mounting. Where authors proposed explanatory frameworks, mistaken identity or indiscriminate mating was the most invoked interpretation, although adaptive functions and socially mediated explanations were also recurrent. Direct measurements of fitness consequences were rare and yielded mixed outcomes, suggesting that the consequences of SSB are context dependent rather than uniformly beneficial or maladaptive. Overall, the available evidence supports the view that SSB in invertebrates is neither anomalous nor reducible to a single mechanism. Instead, it appears as a heterogeneous component of the behavioral repertoire whose expression is shaped by mating system, study type, and taxonomic history.

## Simple Summary

Same-sex sexual behavior (SSB) in invertebrates has often been treated as anecdotal, accidental, or evolutionarily peripheral. By synthesizing published evidence across more than 200 species, this study shows that reported SSB is taxonomically widespread but highly uneven across lineages and study types. The available literature is dominated by insects and by particular kinds of study designs, indicating that current patterns reflect research effort as much as biology. At the same time, the behaviors themselves range from courtship and mounting to copulation, tandem running and, in a separate comparative category, same-role or SSB-like interactions in simultaneous hermaphrodites. Rather than supporting a single explanatory model, the published evidence points to multiple, non-exclusive processes, including indiscriminate mating, socially mediated expression, and context-dependent adaptive benefits. This synthesis places invertebrates more firmly within the comparative study of sexual behavior and highlights where future ethological work is most needed.

## 1. Introduction

Same-sex sexual behavior (SSB), encompassing courtship, mounting, copulation, and pair bonding between individuals of the same sex, has been documented across a broad range of animal taxa, including invertebrates [1,2]. Historically, sexual behavior has often been interpreted through a reproduction-centered framework in which male–female mating and offspring production has traditionally provided the main benchmark for evaluating direct fitness consequences. Within that perspective, same-sex interactions were frequently regarded as incidental, maladaptive, or behaviorally aberrant. Yet the accumulated literature suggests that SSB is far more diverse and context dependent than such a framework implies [3,4].

Invertebrates are particularly informative for evaluating SSB because they combine extraordinary diversity in mating systems, sexual morphology, social organization, and sensory ecology [5]. At the same time, invertebrate studies have contributed substantially to the development and testing of hypotheses on SSB, but this evidence remains scattered across different taxa, study systems, and explanatory frameworks. This imbalance has limited broader comparative inference and has also obscured the possibility that similar behavioral outcomes may arise from different proximate and evolutionary routes across lineages.

The literature generally invokes three broad, non-mutually exclusive explanatory frameworks. The first is mistaken identity or indiscriminate mating, in which same-sex interactions occur when sex discrimination is weak or when the cost of rejecting a potential mate exceeds the cost of occasional mismatch [6,7,8]. The second emphasizes socially or environmentally mediated expression, for example through density, sex-ratio bias, partner scarcity, infection, or predation risk [9,10,11]. The third framework considers adaptive functions, including mating practice, reproductive assurance, anti-predator benefits, or maintenance of social cohesion [2,12,13]. Importantly, these frameworks often overlap rather than compete. Even apparently indiscriminate mating can be favored under ecological conditions in which missed mating opportunities are especially costly [14].

Simultaneous hermaphrodites add an important but conceptually delicate dimension to the study of same-sex sexual behavior. Because each individual combines male and female reproductive functions, sexual interactions in these systems cannot be interpreted through the same binary framework used for gonochoric species [15,16]. Behaviors such as reciprocal mating, sperm donation, hypodermic insemination, or sex-role negotiation may resemble SSB superficially, but they often arise from sex allocation, sexual conflict, reproductive assurance, or partner availability rather than from interactions between individuals occupying equivalent sex categories [17,18,19]. For this reason, hermaphroditic taxa are best treated not as direct equivalents of male–male or female–female interactions, but as a comparative framework for evaluating how flexible reproductive roles can generate sexual behaviors that challenge simple reproductive classifications.

A previous synthesis focused specifically on insects and arachnids [3], but the broader invertebrate literature remains dispersed across many taxonomic groups and study designs. Here, we compile and analyze published evidence of SSB across invertebrates to characterize its reported taxonomic distribution, behavioral diversity, ecological context, and explanatory frameworks. By linking reported behaviors to taxonomic group, study type, and author interpretation, this synthesis helps distinguish broad biological patterns from structural biases in research effort and study design.

## 2. Materials and Methods

SSB was defined according to the observed behavioral phenotype, rather than sexual orientation or preference, which are generally inappropriate or unmeasurable constructs in invertebrates [2,4]. Accordingly, we considered SSB to include courtship, mounting, copulation, genital contact, pair formation, or other sexual behaviors occurring between individuals of the same sex. For simultaneous hermaphrodites, interactions were not classified as SSB simply because both partners possess male and female reproductive functions. These cases were retained only when the reported behavior involved same-role sexual interactions, role conflict, reciprocal role symmetry, or SSB-like interactions explicitly discussed by the original authors as relevant to same-sex or non-standard sexual behavior. Conversely, interactions in which one individual clearly acted as sperm donor and the other as sperm recipient, resulting in fertilization through the female function, were not treated as SSB sensu stricto. This study was conducted following the Preferred Reporting Items for Systematic Reviews and Meta-Analyses (PRISMA) guidelines [20]. The literature search, screening, and selection process were performed in accordance with these recommendations, and a flow diagram summarizing the study selection process is provided in Figure S1. This review was not pre-registered.

Relevant studies were identified through Web of Science, Scopus, ScienceDirect, SpringerLink, and PubMed using combinations of terms such as “same-sex sexual behavior”, “homosexual behavior”, “same-sex courtship”, and taxonomic identifiers including “arthropods”, “mollusks”, “annelids”, and “flatworms”. Searches were conducted between June and September 2025 without language or publication-year restrictions. These search strings were complemented by backward and forward citation tracking using key review papers, particularly Scharf and Martin [3] and Bagemihl [1], to retrieve primary studies in which SSB had been reported incidentally rather than as the main topic.

The initial search yielded 842 records. After removal of duplicates (*n* = 167), 675 titles and abstracts were screened, of which 281 reports were retained for full-text evaluation. Forty-seven reports could not be retrieved. The final analytical dataset comprised 222 primary studies, while 12 reviews were retained only for source tracing or conceptual context. The screening process is summarized in Supplementary Figure S1.

From each primary source we extracted taxonomic information (phylum, order, species), behavioral type, study type, sexual system, and any interpretation advanced by the original authors. Behavioral types were coded from the descriptions provided in the original studies and grouped into broad categories: courtship, including displays, antennation, acoustic or vibrational signaling, or other pre-copulatory behaviors; mounting, when one individual climbed onto or positioned itself over a same-sex partner without clear evidence of genital contact; copulation-like interactions, including genital contact, attempted intromission, copulation, pseudocopulation, insemination, or spermatophore transfer; pairing/tandem behavior, including prolonged association, tandem running, or coordinated pair formation; and attraction/aggregation, when same-sex individuals showed chemically or behaviorally mediated attraction or clustering in a reproductive context. Where available, we also recorded whether fitness consequences were assessed and, if so, whether reported effects were positive, negative, neutral, or indeterminate.

Author interpretations were subsequently grouped into three broad, non-mutually exclusive explanatory frameworks. Records were classified as mistaken identity or indiscriminate mating when the original authors attributed SSB to weak sex discrimination, ambiguous sexual cues, high encounter rates, or mate-recognition rules in which occasional same-sex interactions were interpreted as recognition errors or tolerated by-products of mating effort. Records were classified as socially or environmentally mediated expression when SSB was linked to density, sex-ratio bias, partner scarcity, prior social experience, infection, predation risk, or other contextual factors affecting behavioral expression. Records were classified as adaptive functions when authors proposed or tested direct or indirect benefits, including mating practice, reproductive assurance, anti-predator benefits, maintenance of social cohesion, sperm competition, or increased future mating success. When a study proposed more than one explanation, all relevant interpretations were recorded, but the primary framework emphasized by the original authors was used for summary analyses. Studies that described SSB without an explicit explanatory interpretation were retained in the descriptive synthesis but were not treated as supporting a specific framework.

Because the available literature is dominated by qualitative or categorical observations rather than standardized effect sizes, our quantitative analyses focused on frequencies, proportional summaries, and association tests rather than formal effect-size synthesis across the full dataset. This limitation is common in behavioral evidence syntheses where reporting standards and study designs vary widely [21,22,23].

### 2.1. SSB Mechanisms

To explore variation in reported patterns, we combined descriptive summaries with comparative analyses across taxonomic and behavioral categories. Associations between categorical variables were evaluated primarily with chi-square tests of independence.

### 2.2. SSB Prevalence

To examine reported prevalence while partially accounting for taxonomic imbalance, we calculated a Relative Reporting Index (RRI) by dividing the number of documented SSB species in each order by its known described species richness and multiplying by 1000. Species-richness estimates were obtained primarily from the Catalogue of Life checklist and cross-checked, where necessary, against published higher-level summaries of animal taxonomic richness [24,25]. This metric standardizes reporting density and should not be interpreted as an estimate of biological prevalence.

### 2.3. SSB Hypotheses Evaluation

To explore whether study type and behavioral category predicted whether authors interpreted a case as adaptive or non-adaptive, we fitted a binomial logistic regression using study type and behavioral type as predictors.

The odds ratio contrasting laboratory and field studies was calculated from a 2 × 2 contingency table using the Haldane–Anscombe correction where needed. This estimate was intended as an interpretable summary of the association between study design and interpretative outcome rather than a direct estimate of biological effects.

For phylogenetic analyses, we evaluated whether the presence of reported SSB exhibited clustering across higher taxa using Pagel’s λ and Blomberg’s K. These analyses were based on a binary matrix across 112 invertebrate orders: 27 orders with documented SSB and 85 representative orders with no reports included to provide broader phylogenetic coverage (Supplementary Tables S1 and S2). The order-level phylogeny was assembled as a synthetic backbone from published relationships among major invertebrate clades and taxonomic orders. Because comparable dated phylogenies are not available for all included invertebrate orders, branch lengths were set to equal length, and the resulting tree was used only to evaluate broad phylogenetic structure in reporting patterns, not to infer evolutionary transitions or ancestral states. The topology and order inclusion are provided in Supplementary Figure S3 and Supplementary Table S2. Because absence of reports cannot be equated with true biological absence, these analyses were treated explicitly as analyses of the distribution of current evidence rather than of the evolutionary distribution of SSB itself.

We also conducted an exploratory unsupervised analysis using Principal Component Analysis (PCA) followed by K-means clustering. Categorical variables included phylum, study type, and behavioral category. These variables were one-hot encoded and standardized before dimensionality reduction. K-means clustering was applied to the PCA coordinates using k = 3, corresponding to three broad descriptive sources of structure in the dataset: laboratory insect records dominated by mounting or courtship, field-based arthropod records dominated by copulation-like behavior, and sparse records from phylogenetically distant taxa. Because the analysis was based on one-hot encoded categorical variables, several records shared identical or near-identical PCA coordinates. Therefore, overlapping points may occur in the visualization when records have the same encoded combination of taxonomic, study-type, and behavioral attributes. For graphical clarity only, slight jitter was applied to the plotted points, without altering the PCA coordinates used for clustering or the cluster assignments. PCA loadings, explained variance, and cluster summaries are provided in Supplementary Table S3.

Statistical analyses were conducted in R v4.3.2 and Python v3.13.5. Chi-square tests, odds ratios, logistic regression, and phylogenetic signal analyses were performed in R. Data cleaning, categorical encoding, PCA, K-means clustering, PCA tables, and PCA visualizations were performed in Python using pandas, NumPy, scikit-learn, matplotlib, and seaborn. Software versions, analytical roles, and package references are provided in Supplementary Table S4.

### 2.4. Sensitivity and Robustness Considerations

To evaluate robustness, we repeated descriptive summaries after excluding anecdotal or weakly contextualized reports and after collapsing repeated records for the same species. These checks did not qualitatively alter the major patterns in the distribution of behavioral categories or explanatory frameworks.

We also considered whether alternative coding decisions might be driving the multivariate patterns, for example by collapsing mounting and copulation or by merging mistaken identity and indiscriminate mating into a single interpretative class. Because these variants produced similar qualitative outcomes, the main conclusions did not depend strongly on one particular coding scheme.

Finally, we considered whether taxonomic imbalance or repeated reporting within a few well-studied species could dominate the dataset. Although some orders and species were clearly overrepresented, SSB was nevertheless documented across multiple phyla, study types, and mating systems. The trends reported below should therefore be interpreted as broad patterns in the published literature, while recognizing that the literature itself remains uneven.

All of these sensitivity checks indicate that the principal conclusions are not contingent upon one particular coding decision or a single overrepresented lineage. Rather, they reflect persistent patterns in the currently available evidence on SSB across invertebrates.

## 3. Results

### 3.1. Reported Distribution of Same-Sex Sexual Behavior Across Invertebrates

**Figure 1:**
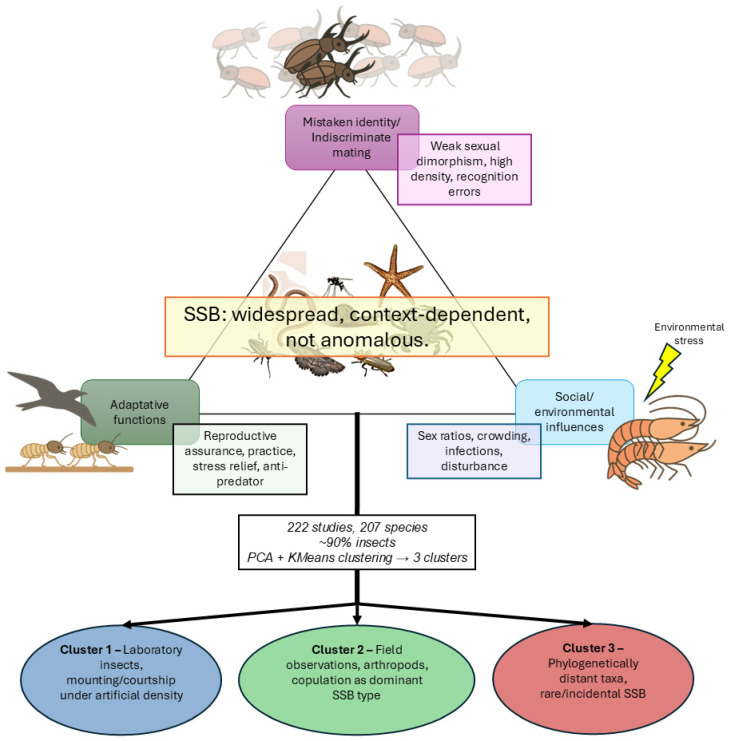
Conceptual synthesis of the main explanatory frameworks proposed to interpret same-sex sexual behavior (SSB) in invertebrates: mistaken identity or indiscriminate mating, socially mediated expression, and adaptive functions. The lower panel summarizes the exploratory clustering analysis and should be interpreted as a visualization of broad structure in the literature rather than as evidence of discrete biological syndromes.

**Figure 2:**
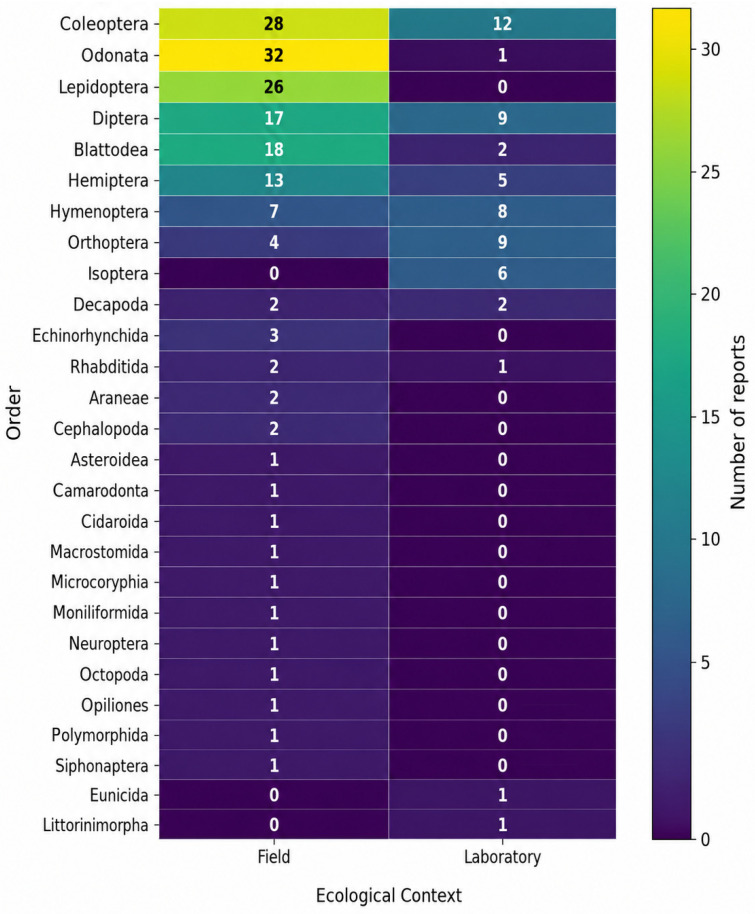
Heatmap of reported same-sex sexual behavior (SSB) across invertebrate orders, separated by study type. Each cell shows the number of published records documenting SSB in field or laboratory settings. Warmer colors indicate greater reporting frequency.

The systematic synthesis identified 222 primary studies documenting same-sex sexual behavior (SSB) in approximately 207 invertebrate species across seven phyla: Arthropoda, Mollusca, Annelida, Echinodermata, Platyhelminthes, Acanthocephala, and Nematoda. The temporal span of the dataset, from 1895 to 2024, indicates that SSB has long been observed in invertebrates, although documentation has accumulated in a markedly uneven way across lineages. Arthropods dominated the literature, and insects alone accounted for approximately 89% of all records (Figure 1). Within insects, the most heavily represented orders were Diptera, Coleoptera, Hymenoptera, and Lepidoptera (Figure 2). A detailed synthesis of all recorded cases, including species identity, behavioral category, study type, and interpretative framework, is provided in Table S1.

This taxonomic concentration was reflected in the overall distribution of reports, which was strongly non-random across major groups (χ^2^ = 950.0, *p* < 0.001; Table 1). However, standardization by species richness altered this picture substantially. The Relative Reporting Index (RRI) revealed that several orders with comparatively few raw reports, such as Cidaroida, Odonata, and Cephalopoda, had disproportionately high reporting intensity, whereas hyper-diverse groups such as Coleoptera and Diptera appeared relatively underrepresented once richness was taken into account (Figure S2).

### 3.2. Study Type and Behavioral Distribution

Most records in the dataset came from field-based studies, although the relative contribution of field and laboratory work differed substantially among orders (Figure 2). Odonata (*n* = 32) and Lepidoptera (*n* = 26) were represented exclusively by field observations, whereas Coleoptera combined both field (*n* = 27) and laboratory (*n* = 11) studies. Diptera also showed a mixed distribution (field = 17, laboratory = 8), while Hymenoptera was comparatively enriched in laboratory-based studies (laboratory = 8, field = 7). Thus, the literature is structured not only by taxon, but also by systematic differences in how mating behavior has been studied.

Copulation-like interactions were the most frequently reported category of SSB, occurring in more than half of the studies and spanning a broad taxonomic range. Courtship was reported in approximately 42% of studies, and mounting in roughly 37%. These behavioral categories were themselves unevenly distributed across the literature. Courtship and mounting were especially common in laboratory studies of Diptera and Hymenoptera, whereas copulation-like interactions dominated field-based reports in Coleoptera, Lepidoptera, and Odonata (Figure 2). In other words, the apparent distribution of behavioral forms depended not only on lineage, but also on study type.

### 3.3. Interpretative Frameworks and Fitness Consequences

Where authors proposed explicit explanatory frameworks, mistaken identity or indiscriminate mating was the most frequently invoked interpretation. These cases were concentrated mainly in insects and other arthropods in which same-sex mounting, courtship, or copulation-like behavior was interpreted as arising from weak sex discrimination, ambiguous cues, or broad mate-searching rules. Adaptive explanations were also frequent and were most often associated with cases in which SSB was linked to measurable or proposed benefits, including reproductive assurance, anti-predator behavior, social cohesion, or competitive interactions. Socially or environmentally mediated interpretations were less frequent overall but were concentrated in systems where density, sex ratio, partner availability, prior social experience, infection, or predation risk were explicitly discussed as triggers of SSB expression. Thus, the three frameworks were not evenly distributed across the literature; mistaken identity and indiscriminate mating were most often associated with mate-recognition constraints, socially mediated explanations with experimentally or demographically variable contexts, and adaptive interpretations with systems in which potential reproductive or survival consequences were proposed. Across the literature as a whole, this apparent balance should be treated cautiously because many studies remained descriptive and did not formally test alternative hypotheses.

Direct evidence for fitness consequences was limited. Only 22 studies explicitly evaluated reproductive or survival-related outcomes, and these yielded mixed results: 10 reported positive effects, six negative effects, and six neutral or inconclusive effects. The available quantitative summary from these cases showed no overall directional effect (Hedges’ g = 0.103; 95% CI = −0.016 to 0.222), suggesting that the consequences of SSB are context dependent rather than uniformly beneficial or detrimental.

### 3.4. Sexual Systems, Study Type, and Interpretation

Strict SSB records were concentrated primarily in gonochoric taxa and most often involved male–male interactions. Hermaphroditic and sex-flexible taxa formed a smaller comparative subset and were more frequently associated with interpretations involving reproductive assurance, sperm allocation, or sex-role flexibility. Because reproductive roles in these taxa do not map directly onto fixed male and female individuals, these records were interpreted cautiously and not treated as direct equivalents of gonochoric SSB. The association between sexual system and interpretative framework was significant (χ^2^ = 16.2, *p* = 0.002; Table 1).

Interpretation also varied strongly with study type. Laboratory studies were much more likely than field studies to frame SSB in adaptive or mechanistic terms, whereas field reports were more often descriptive. The odds ratio contrasting laboratory and field studies was high (OR = 123.0; 95% CI = 30.4–501.6; Table 1). This extremely large effect size likely reflects structural differences in study design and reporting rather than a biologically meaningful contrast. Logistic regression likewise suggested a weak trend for copulation-type reports to be more often interpreted as adaptive (z = 1.92, *p* = 0.055; Table 1), although this effect fell just short of conventional significance.

### 3.5. Phylogenetic and Exploratory Multivariate Structure

**Figure 3:**
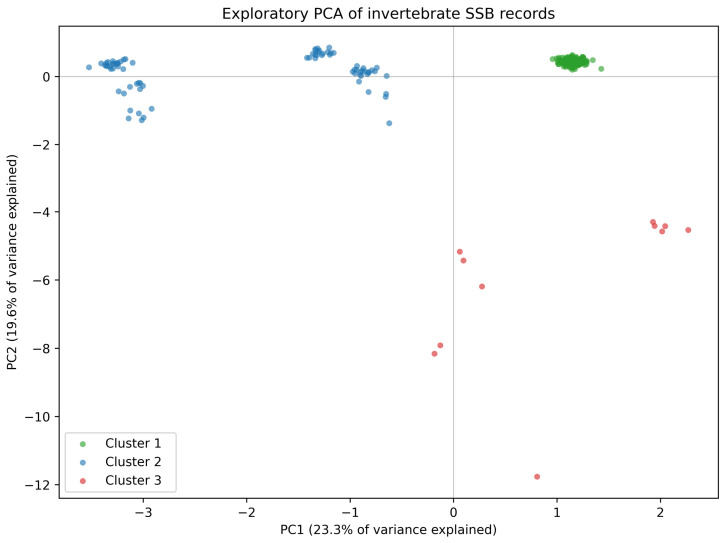
Exploratory Principal Component Analysis (PCA) of invertebrate same-sex sexual behavior (SSB) reports. Each point represents a strict SSB record classified by taxonomic group, study type, and behavioral category after one-hot encoding and standardization of categorical variables. Colors indicate the three K-means groupings used to summarize broad co-occurrence patterns in the dataset.

The exploratory PCA followed by K-means clustering recovered three broad groupings in the literature (Figure 3). After excluding records retained only as comparative, non-strict SSB cases, the PCA was conducted on 217 strict SSB records. The first two principal components explained 39.4% of the total variance (PC1 = 23.3%; PC2 = 19.6%). PC1 was driven mainly by positive loadings for field records and copulation-like interactions and negative loadings for laboratory records, courtship, mounting, and pairing/tandem categories. This axis therefore broadly separated field-based copulation-like reports from laboratory-based courtship, mounting, or pairing reports. PC2 was driven primarily by a strong positive loading for Arthropoda and negative loadings for sparsely represented non-arthropod groups, including acanthocephalans, echinoderms, mollusks, and annelids. This axis therefore mainly reflected the strong taxonomic concentration of the dataset in arthropods rather than a distinct biological gradient. Because these two axes explained a modest proportion of the total variance, the PCA plot should be interpreted as a simplified two-dimensional visualization of broad co-occurrence patterns rather than as a complete representation of the multivariate structure of the dataset. The three K-means groupings should consequently be interpreted as descriptive summaries of how taxonomic focus, study type, and behavioral category co-occur in the published literature, not as evidence of discrete ethological syndromes or evolutionary routes. PCA loadings are provided in Supplementary Table S4.

## 4. Discussion

This synthesis confirms that same-sex sexual behavior (SSB) has been documented across a wide range of invertebrate taxa, but it also shows that the apparent distribution of SSB is strongly shaped by research effort. The dominance of insect records likely reflects their accessibility, the long tradition of studying insect mating systems, and the relative ease with which many insect species can be observed or manipulated in laboratory settings, rather than a true biological concentration of SSB in insects alone. This interpretation is consistent with broader concerns in behavioral ecology that published patterns often reflect uneven observational effort, taxonomic familiarity, and methodological accessibility as much as the biological distribution of the trait itself [21,22,23].

A central implication of this pattern is that the absence of reports should not be interpreted as evidence that SSB is absent. Many invertebrate lineages, especially marine, benthic, parasitic, and soft-bodied taxa, remain poorly characterized behaviorally. This is particularly important because several non-insect groups included in our dataset, such as cephalopods, echinoderms, annelids, nematodes, and acanthocephalans, show that SSB or SSB-like interactions can occur outside the insect systems that dominate the literature [3,26,27,28]. Thus, current taxonomic patterns should be viewed primarily as patterns of reported evidence, not as estimates of true prevalence.

The association between study type and interpretation further highlights the influence of methodology on inference. Laboratory studies were more likely to invoke adaptive or process-oriented explanations, whereas field reports were often brief and descriptive. This asymmetry does not imply that adaptive forms of SSB are intrinsically more common in laboratory settings. Rather, laboratory studies are more often designed to manipulate social context, density, sex ratio, sensory cues, or prior experience, allowing authors to test specific mechanisms that field observations alone cannot easily separate [6,29,30]. The large odds ratio reported should therefore be read in this methodological light, not as a biological estimate of how strongly study setting determines adaptive value.

The predominance of mistaken identity or indiscriminate mating as an explanatory framework also requires a nuanced interpretation. In many systems, these terms should not be treated simply as synonyms for maladaptive error. Reduced mate discrimination may itself be favored when the cost of missing a mating opportunity exceeds the cost of occasional same-sex interactions, especially in dense aggregations, male-biased assemblages, short-lived adults, or situations where mate encounters are brief and unpredictable [2,7,8]. The water bug *Palmacorixa nana* (Hemiptera: Corixidae) provides a useful example: males frequently mount other males, but this appears to arise from a size-based mate-searching rule because females are larger than males; same-sex mounting may therefore represent a tolerable inefficiency within a broader mate-location strategy rather than a purely pathological behavior [31]. Similarly, in *Teleogryllus oceanicus* (Orthoptera: Gryllidae), males appear to relax sex discrimination under social conditions suggesting female availability, which supports the idea that SSB can emerge from plastic adjustment of mating thresholds rather than from a fixed inability to recognize sex [6]. This interpretation also helps place our results in relation to the insect- and arachnid-focused synthesis of Scharf and Martin [3], which emphasized mistaken identity as a common explanation for same-sex interactions. Our broader dataset supports the relevance of mate-recognition constraints, but suggests that these cases are better viewed as part of a continuum ranging from sensory error to adaptive relaxation of mating thresholds. In some insects, male–male interactions may also be facilitated when the mounted male expresses female-like cues or behaviors, or when males exploit female mimicry in competitive contexts, as reported in rove beetles and other arthropod systems [3,32]. Such cases do not fit neatly into a simple “error versus adaptation” dichotomy: they may reflect recognition ambiguity, opportunistic mating rules, or sexually selected strategies depending on the ecological and social context.

Social and ecological context appears especially important in explaining why the same behavioral category may have different meanings across taxa. In termites, same-sex tandem running and female–female colony foundation have been interpreted in relation to mate limitation, predation risk, and reproductive assurance. In *Reticulitermes speratus* (Blattodea: Rhinotermitidae), females that fail to pair with males can found colonies alone or in female–female pairs, and female–female cooperation increases survivorship relative to solitary foundation, even though reproductive output per female may be lower than in male–female pairs [9]. Such cases show that same-sex interactions can become functional under specific demographic or ecological constraints, particularly when the alternative is failure to establish a colony. These findings contrast with systems in which SSB appears mainly as a sensory or recognition error, emphasizing that similar behaviors may arise through different evolutionary routes.

At the same time, not all cases should be forced into an adaptive interpretation. Several studies show that SSB can carry measurable costs, including reduced longevity, wasted reproductive effort, physical damage, or reduced time available for heterosexual mating [3,26,33,34]. In acanthocephalan worms, for instance, male–male copulatory behavior can result in cement caps that block the genital region of the male victim and may remove him from the reproductive population, a case interpreted in the context of sexual selection and male–male competition [26]. In parasitic wasps, male–male sexual interactions have been associated with longevity costs, although the persistence of the behavior suggests that costs may be context dependent or counterbalanced by other benefits [34]. These examples reinforce the conclusion that SSB cannot be classified as uniformly adaptive or uniformly maladaptive.

Viewed across taxonomic and ecological contexts, the three explanatory frameworks appear to be associated with different kinds of systems rather than with mutually exclusive biological categories. Mistaken identity or indiscriminate mating was most evident in arthropods, particularly insects, where rapid mate searching, weak or overlapping sexual cues, high encounter rates, or low costs of brief mating attempts can favor broad mate-recognition thresholds. Socially or environmentally mediated SSB was most apparent in systems where behavioral expression depends strongly on local conditions, such as density, operational sex ratio, partner availability, prior social experience, infection, or predation risk. These cases suggest that SSB may often be expressed plastically rather than as a fixed behavioral trait. Adaptive interpretations were more common in taxa or ecological settings where same-sex interactions could plausibly affect survival, reproductive assurance, social cohesion, or competitive outcomes, including termites, some hymenopterans and hemipterans, acanthocephalans, and hermaphroditic or sex-flexible systems. Therefore, taxonomic group alone does not predict the interpretation of SSB; rather, the relevant predictors appear to be mating-system architecture, cue reliability, encounter rate, demographic constraint, and the ecological cost of rejecting or accepting potential partners.

Hermaphroditic taxa require particular caution. In systems where sperm transfer fertilizes the female function of the partner, the interaction is better interpreted as different-sex function at the gametic or reproductive-role level, even if both individuals are morphologically hermaphroditic. Therefore, such cases should do not represent a direct evidence of SSB sensu stricto. Their value in this review is comparative: they illustrate how behaviors that superficially resemble SSB in gonochoric species may instead arise from sex allocation, sperm competition, reciprocal role conflict, or reproductive assurance [16,17,35], allowing hermaphrodites to inform broader questions about how sexual roles, partner availability, and reproductive conflict shape sexual behavior.

The phylogenetic and multivariate results should also be interpreted conservatively. The detected phylogenetic structure likely reflects both biological clustering and the uneven distribution of behavioral research across invertebrate lineages. Because many orders remain unsampled or weakly sampled, phylogenetic signal in reported SSB cannot be equated with phylogenetic signal in the true occurrence of SSB. Similarly, the exploratory PCA and K-means clustering summarize how taxonomic focus, study type, and behavioral categories co-occur in the published literature, but they should not be interpreted as discrete ethological syndromes or evolutionary routes. This caution is further supported by the modest proportion of variance explained by the first two PCA axes, which indicates that the two-dimensional PCA plot captures only part of the multivariate structure of the dataset. Therefore, the PCA should be understood as a simplified visualization of broad co-occurrence patterns rather than as a complete representation of the variation in SSB records. This limitation is especially relevant because categorical behavioral datasets are sensitive to coding decisions, uneven taxonomic sampling, and repeated study of a small number of model systems [21,22,23].

Overall, our results support a pluralistic interpretation of SSB in invertebrates. Some cases are best explained by weak sex discrimination or mate-recognition constraints; others by social experience, density, sex ratio, or partner limitation; and a smaller number by direct or indirect adaptive benefits. This conclusion is consistent with previous reviews arguing that SSB is evolutionarily heterogeneous and should not be reduced to a single mechanism [2,3,4]. The main contribution of the present synthesis is to show that this heterogeneity extends broadly across invertebrates, while also making clear that the available evidence remains strongly structured by taxonomic and methodological bias. In this context, the three descriptive clusters recovered by the PCA/K-means analysis are best understood as a practical guide to different evidence gaps rather than as discrete ethological syndromes. Laboratory-dominated insect records, especially those involving courtship or mounting, would benefit from experimental designs that explicitly manipulate density, sex ratio, prior social experience, and sensory cues while measuring downstream reproductive consequences. Field-based arthropod records dominated by copulation-like interactions require more standardized reporting of population context, operational sex ratio, encounter rate, individual condition, interaction duration, and measurable costs or benefits. In contrast, sparse records from phylogenetically distant taxa, including mollusks, echinoderms, annelids, nematodes, and acanthocephalans, still require basic behavioral description, repeated observations, and clearer separation between strict same-sex interactions and superficially similar behaviors associated with hermaphroditism, sex-role flexibility, or reproductive conflict.

Future work should therefore move beyond simply documenting the occurrence of SSB toward testing explicit alternatives within the ecological and methodological context in which each type of record is produced. Comparative syntheses will be strengthened by studies that combine standardized behavioral observations with manipulations of social environment, mate availability, sensory information, and ecological risk, while measuring reproductive or survival consequences directly. Such work is needed to distinguish among mistaken identity, adaptive indiscriminate mating, socially mediated plasticity, and direct fitness benefits. Until such evidence accumulates, SSB in invertebrates is best understood as a heterogeneous and context-dependent component of the behavioral repertoire, shaped by mating system architecture, sensory ecology, ecological constraint, and study design.

## Figures and Tables

**Table 1:** Summary of main statistical analyses evaluating taxonomic, study-type, and behavioral predictors of same-sex sexual behavior (SSB) across invertebrates.

Analysis	Variable Tested	Test	Statistic	*p*-Value	Interpretation
Chi-square	Major taxonomic group × reported SSB cases	χ^2^	950.0	<0.001	Strongly non-random taxonomic distribution of reports
Chi-square	Sexual system × interpretative framework	χ^2^	16.2	0.002	Hermaphroditic taxa more often associated with adaptive interpretations
Odds ratio	Study type (Laboratory vs. Field)	OR	123.0 (95% CI 30.4–501.6)	—	Laboratory studies more often interpret SSB as adaptive; estimate should be read cautiously as a feature of study design
Logistic regression	Behavioral type × adaptive interpretation	z	1.92	0.055	Weak trend toward adaptive interpretation in copulation-type reports

## Data Availability

All data generated or analyzed during this study are included in this published article and in the Supplementary Materials.

## Supplementary Materials

The following supporting information can be downloaded at https://www.mdpi.com/article/10.3390/insects17060611/s1 [36,37,38,39,40,41,42,43,44,45,46,47,48,49,50,51,52,53,54,55,56,57,58,59,60,61,62,63,64,65,66,67,68,69,70,71,72,73,74,75,76,77,78,79,80,81,82,83,84,85,86,87,88,89,90,91,92,93,94,95,96,97,98,99,100,101,102,103,104,105,106,107,108,109,110,111,112,113,114,115,116,117,118,119,120,121,122,123,124,125,126,127,128,129,130,131,132,133,134,135,136,137,138,139,140,141,142,143,144,145,146,147,148,149,150,151,152,153,154,155,156,157,158,159,160,161,162,163,164,165,166,167,168,169,170,171,172,173,174,175,176,177,178,179,180,181,182,183,184,185,186,187,188,189,190,191], Table S1: Summary of documented cases of same-sex sexual behavior (SSB) across invertebrates; Table S2: List of 85 Orders with No Reported SSB used to explore phylogenetic signals in the evolutionary conservatism of SSB; Table S3: PCA loadings for PC1 and PC2; Table S4: Software and computational packages used in the analytical workflow; Table S5: PRISMA_2020_checklist; Figure S1: PRISMA flow diagram summarizing the identification, screening, eligibility assessment, and inclusion of studies in the systematic review; Figure S2: Relative Reporting Index (RRI × 1000) for SSB across 24 invertebrate orders; Figure S3: Synthetic order-level phylogenetic backbone used for exploratory phylogenetic signal analyses.
