# Supplementary material for: Same-Sex Sexual Behavior in Invertebrates: A Systematic Synthesis of Prevalence, Study Type, and Interpretation
Source: Insects. 2026 Jun 10;17(6):611. doi: 10.3390/insects17060611 (PMC13299977; doi:10.3390/insects17060611)
Supplement: Supplementary file 1 [file insects-17-00611-s001.zip › insects-4294360-supplementary/insects-4294360-supplementary-Final/Table S1-S4, Figure S1-S3.pdf]

Supplementary material

Table S1 Summary of documented cases of same-sex sexual behavior (SSB) across invertebrates. Each record includes the phylum, order, and species, type of sexual behavior observed, ecological context (field or laboratory), main interpretation proposed by original authors to explain the behavior, reported fitness effects and full reference. This dataset compiles published evidence illustrating the diversity of taxa and contexts in which SSB has been reported

Phylum	Order	Species	Sexual Behavior	Context	Interpretation proposed	Fitness effect reported	Reference(s)
Arthropoda	Araneae	*Doryonychus raptor*	Male-male copulation	Field	None	None	[36]
Arthropoda	Araneae	*Portia fimbriata*	Male-male and female-female copulation	Field	None	None	[37]
Echinodermata	Asteroidea	*Archaster angulatus*	Male-male pairing	Field	Indiscriminate mating	None	[38]
Arthropoda	Blattodea	*Blaberus, Archimandrita, Byrsotria*	Male-male courtship	Laboratory	Adaptive	Positive	[39]
Arthropoda	Blattodea	*Blatta orientalis*	Male-male courtship	Field	None	None	[40]
Arthropoda	Blattodea	*Blattella germanica*	Male-male courtship	Field	None	None	[41]
Arthropoda	Blattodea	*Byrsotria fumigata*	Male-male copulation	Field	None	None	[42]
Arthropoda	Blattodea	*Byrsotria fumigata*	Male-male courtship	Field	None	None	[42]
Arthropoda	Blattodea	*Eublaberus distanti (Cockroaches)*	Male-male courtship	Laboratory	None	None	[43]
Arthropoda	Blattodea	*Eurycotis floridana*	Male-male courtship	Field	None	None	[44]
Arthropoda	Blattodea	*Henchoustedenia flexivitta*	Male-male copulation	Field	None	None	[45]
Arthropoda	Blattodea	*Latiblattella angustifrons*	Male-male courtship	Field	None	None	[46]
Arthropoda	Blattodea	*Leucophaea maderae*	Male-male courtship	Field	None	None	[47]

Arthropoda	Blattodea	*Nauphoeta cinerea*	Male-male courtship	Field	None	None	[47]
Arthropoda	Blattodea	*Nauphoeta cinerea*	Male-male copulation	Field	None	None	[45]
Arthropoda	Blattodea	*Parcoblatta fulvescens*	Male-male courtship	Field	None	None	[48]
Arthropoda	Blattodea	*Periplaneta americana*	Male-male copulation	Field	None	None	[40]
Arthropoda	Blattodea	*Periplaneta australasiae*	Male-male courtship	Field	None	None	[40]
Arthropoda	Blattodea	*Periplaneta brunnea*	Male-male courtship	Field	None	None	[40]
Arthropoda	Blattodea	*Periplaneta fuliginosa*	Male-male copulation	Field	None	None	[40]
Arthropoda	Blattodea	*Periplaneta japonica*	Male-male courtship	Field	None	None	[40]
Arthropoda	Blattodea	*Petasodes dominicana*	Male-male copulation	Field	None	None	[49]
Arthropoda	Blattodea	*Supella longipalpa*	Male-male courtship	Field	None	None	[50]
Echinodermata	Camarodonta	*Lytechinus variegatus*	Male-male pairing	Field	Mistaken identity	None	[51]
Mollusca	Cephalopoda	*Deep-sea squid spp.*	Male-male mounting	Field	Indiscriminate mating	None	[28]
Mollusca	Cephalopoda	*Dosidicus gigas (Humboldt squid)*	Male-male copulation	Field	Indiscriminate mating	None	[52]
Echinodermata	Cidaroida	*Stylocidaris lineata*	Male-male aggregations for reproduction	Field	Adaptive	None	[53]
Arthropoda	Coleoptera	*Acanthoscelides obtectus*	Male-male and female-female mounting	Lab	Mistaken identity	Negative	[54]
Arthropoda	Coleoptera	*Aclees sp. cf. foveatus*	Male-male mounting	Field	None	None	[55]
Arthropoda	Coleoptera	*Aegus chelifer chelifer*	Male-male mounting	Lab	Mistaken identity	None	[56]
Arthropoda	Coleoptera	*Aleochara curtula*	Male-male and male-to-female	Field	None	None	[57]

			transvestism copulation				
Arthropoda	Coleoptera	*Antitrogus consanguineus*	Male-male copulation	Field	None	None	[58]
Arthropoda	Coleoptera	*Bolitotherus cornutus*	Male-male copulation	Laboratory	Adaptive	None	[59]
Arthropoda	Coleoptera	*Callosobruchus chinensis*	Female-female copulation	Field	None	None	[60]
Arthropoda	Coleoptera	*Callosobruchus maculatus*	Male and female same-sex mounting	Laboratory / Field	Adaptive	Variable and complex	[61]
Arthropoda	Coleoptera	*Ceruchus sp.*	Male-male copulation	Field	None	None	[62]
Arthropoda	Coleoptera	*Cotalpa lanigera*	Male-male copulation	Field	None	None	[63]
Arthropoda	Coleoptera	*Cyclocephala distincta*	Male-male courtship	Lab	Mistaken identity	None	[64]
Arthropoda	Coleoptera	*Cyclocephala immaculata*	Male-male copulation	Field	None	None	[63]
Arthropoda	Coleoptera	*Diaprepes abbreviatus*	Female-female mounting	Lab	Mistaken identity	Negative	[65]
Arthropoda	Coleoptera	*Diaprepes abbreviatus*	Female-female copulation	Field	None	None	[66]
Arthropoda	Coleoptera	*Dorcus sp*	Male-male copulation	Field	None	None	[62]
Arthropoda	Coleoptera	*Euscepes postfasciatus (Weevil)*	Male-male copulation	Laboratory	Mistaken identity	None	[67]
Arthropoda	Coleoptera	*Gnatocerus cornutus*	Male-male mounting	Laboratory	Adaptive	Positive	[68]
Arthropoda	Coleoptera	*Hypera postica*	Female-female copulation	Field	None	None	[69]
Arthropoda	Coleoptera	*Leistotrophus versicolor*	Male-male and male-to-female transvestism copulation	Field	None	None	[32]

Arthropoda	Coleoptera	*Lucanus sp*	Male-male copulation	Field	None	None	[62]
Arthropoda	Coleoptera	*Macrodactylus subspinosa*	Male-male copulation	Field	None	None	[70]
Arthropoda	Coleoptera	*Meloe angusticollis*	Male-male copulation	Field	None	None	[71]
Arthropoda	Coleoptera	*Meloe dianella*	Male-male copulation	Field	None	None	[71]
Arthropoda	Coleoptera	*Meloe proscarabaeus*	Male-male and female-female copulation	Field	None	None	[72]
Arthropoda	Coleoptera	*Meloe violaceus*	Male-male and female-female copulation	Field	None	None	[72]
Arthropoda	Coleoptera	*Melolontha vulgaris*	Male-male copulation	Field	None	None	[73,74]
Arthropoda	Coleoptera	*Nicrophorus spp*	Male-male copulation	Laboratory	Adaptive	None	[13]
Arthropoda	Coleoptera	*Otiorrhynchus pupillatus*	Female-female copulation	Field	None	None	[75]
Arthropoda	Coleoptera	*Pelidnota punctata*	Male-male copulation	Field	None	None	[63]
Arthropoda	Coleoptera	*Phoracantha semipunctata*	Female-female copulation	Field	None	None	[76]
Arthropoda	Coleoptera	*Platycerus sp.*	Male-male copulation	Field	None	None	[62]
Arthropoda	Coleoptera	*Polyphylla hammondi*	Male-male copulation	Field	None	None	[63]
Arthropoda	Coleoptera	*Popillia japonica*	Male-male mounting	Field	Indiscriminate mating	Negative	[77]
Arthropoda	Coleoptera	*Popillia japonica*	Male-male and female-female copulation	Field	None	None	[78,79]
Arthropoda	Coleoptera	*Tribolium castaneum*	Male-male copulation	Lab	Indiscriminate mating	None	[80]
Arthropoda	Coleoptera	*Tribolium castaneum*	Male-male copulation	Lab	Social context	None	[30]
Arthropoda	Coleoptera	*Tribolium castaneum*	Male-male copulation	Lab	Mistaken identity	Negative	[7]

Arthropoda	Coleoptera	*Tribolium castaneum*	Male-male copulation	Field	None	None	[81,82]
Arthropoda	Coleoptera	*Xylotrechus pyrrhoderus*	Female-female copulation	Field	None	None	[83]
Arthropoda	Decapoda	*Calappa lophos*	Male-male copulation	Lab	Social context	None	[84]
Arthropoda	Decapoda	*Calappa lophos*	Male-male copulation	Field	None	None	[84]
Arthropoda	Decapoda	*Lysmata wurdemanni*	Male-male courtship	Field and lab	Adaptive	None	[85]
Arthropoda	Diptera	*Ceratitis capitata*	Male-male and female-female copulation	Field	None	None	[86,87]
Arthropoda	Diptera	*Cochliomyia homnivorax*	Male-male and female-female copulation	Field	None	None	[88]
Arthropoda	Diptera	*Dolichopus popularis*	Male-male copulation	Field	None	None	[89]
Arthropoda	Diptera	*Drosophila affinis*	Male-male copulation	Field	None	None	[90]
Arthropoda	Diptera	*Drosophila melanogaster*	Male-male courtship	Lab	Social context	None	[91]
Arthropoda	Diptera	*Drosophila melanogaster*	Male-male courtship	Lab	Adaptive	None	[92]
Arthropoda	Diptera	*Drosophila melanogaster*	Male-male courtship	Lab	Mistaken identity	None	[93]
Arthropoda	Diptera	*Drosophila melanogaster*	Male-male courtship	Lab	Adaptive	None	[94]
Arthropoda	Diptera	*Drosophila melanogaster*	Male-male courtship	Review	Adaptive	Negative	[95]
Arthropoda	Diptera	*Drosophila melanogaster*	Male-male copulation and courtship	Lab	Adaptive	Negative	[96]
Arthropoda	Diptera	*Drosophila melanogaster*	Male-male and female-female copulation	Field	None	None	[97]
Arthropoda	Diptera	*Euarestoides acutangulus*	Male-male copulation	Field	None	None	[98]
Arthropoda	Diptera	*Euleia fratria*	Male-male copulation	Field	None	None	[99]

Arthropoda	Diptera	*Fannia femoralis*	Male-male copulation	Field	None	None	[100]
Arthropoda	Diptera	*Fucellia maritima*	Male-male copulation	Field	None	None	[90]
Arthropoda	Diptera	*Fucomyia frigida*	Male-male copulation	Field	None	None	[90]
Arthropoda	Diptera	*Glossina morsitans*	Male-male copulation	Field	None	None	[101]
Arthropoda	Diptera	*Hermetia illucens (Black soldier fly)*	Male-male courtship	Lab	Mistaken identity	None	[102]
Arthropoda	Diptera	*Hypoderma tarandi*	Male-male copulation	Field	None	None	[103]
Arthropoda	Diptera	*Leucopis palumbii*	Male-male courtship	Lab	Indiscriminate mating	None	[104]
Arthropoda	Diptera	*Medetera spp*	Male-male copulation	Field	None	None	[105]
Arthropoda	Diptera	*Musca domestica*	Male-male copulation	Field	None	None	[101]
Arthropoda	Diptera	*Protophormia terrae-novae*	Male-male copulation	Field	None	None	[106]
Arthropoda	Diptera	*Scatella sp.*	Male-male copulation	Field	None	None	[90]
Arthropoda	Diptera	*Sphyracephala detrahens*	Male-male and female-female courtship	Lab	Mistaken identity	None	[107]
Arthropoda	Diptera	*Stictochironomus crassiforceps*	Male-male copulation	Field	None	None	[108,109]
Acanthocephala	Echinorhynchida	*Acanthocephalus parksidei*	Male-male copulation	Field	None	None	[26]
Acanthocephala	Echinorhynchida	*Echinorhynchus truttae*	Male-male copulation	Field	None	None	[26]
Acanthocephala	Echinorhynchida	*Moniliformis moniliformis*	Male-male mounting	Field	Indiscriminate mating	Negative	[26]
Annelida	Eunicida	*Ophryotrocha diadema*	Male-male copulation	Lab	Mistaken identity	None	[110]
Arthropoda	Hemiptera	*Afrocimex sp.*	Male-male copulation	Field	None	None	[111,112]
Arthropoda	Hemiptera	*Alloeorhynchus furens*	Male-male copulation	Field	None	None	[111]
Arthropoda	Hemiptera	*Ambrysus occidentalis*	Male-male copulation	Field	None	None	[113]

Arthropoda	Hemiptera	*Bactericera cockerelli*	Male-male courtship	Lab	Indiscriminate mating	None	[114]
Arthropoda	Hemiptera	*Callosobruchus maculatus*	Male-male and female-female mounting	Lab	Adaptive	None	[115]
Arthropoda	Hemiptera	*Cimex lectularius*	Male-male copulation	Field	None	None	[111,112]
Arthropoda	Hemiptera	*Cimex lectularius (bed bugs)*	Male-male copulation	Lab	Mistaken identity	Negative	[116]
Arthropoda	Hemiptera	*Embiophila sp.*	Male-male copulation	Field	None	None	[111,112]
Arthropoda	Hemiptera	*Gerris lacustris (Water strider)*	Male-male copulation	Lab	Social context	None	[117]
Arthropoda	Hemiptera	*Hesperoctenes sp.*	Male-male copulation	Field	None	None	[111]
Arthropoda	Hemiptera	*Latrocimex spectans*	Male-male copulation	Field	None	None	[111]
Arthropoda	Hemiptera	*Limnoportus notabilid*	Male-male copulation	Field	None	None	[118]
Arthropoda	Hemiptera	*Limnoporus dissortis*	Male-male copulation	Field	None	None	[118]
Arthropoda	Hemiptera	*Nezara viridula*	Male-male copulation	Field	None	None	[119]
Arthropoda	Hemiptera	*Oncopeltus fasciatus*	Male-male copulation	Field	None	None	[120]
Arthropoda	Hemiptera	*Palmacorixa nana*	Male-male copulation	Field	None	None	[31]
Arthropoda	Hemiptera	*Tenagogerris euphrosyne*	Male-male copulation	Lab	Adaptive	Positive	[121]
Arthropoda	Hemiptera	*Xylocoris maculipennis*	Male-male copulation	Field	None	None	[111]
Arthropoda	Hymenoptera	*Aethina tumida*	Male-male copulation	Lab	Social context	None	[122]
Arthropoda	Hymenoptera	*Aphidius colemani*	Male-male courtship and mounting	Lab	None	None	[112,123]
Arthropoda	Hymenoptera	*Aphidius colemani*	Male-male mounting, antennal rubbing	Lab	Indiscriminate mating	None	[112,124]

Arthropoda	Hymenoptera	*Aphidius ervi*	Male-male copulation	Lab	None	None	[112,125]
Arthropoda	Hymenoptera	*Centris pallida*	Male-male copulation	Field	None	None	[126]
Arthropoda	Hymenoptera	*Cortesia rubecula*	Male-male and female-female copulation	Field	None	None	[127]
Arthropoda	Hymenoptera	*Diaeretiella rapae*	Male-male copulation	Field	Social context	None	[112,128]
Arthropoda	Hymenoptera	*Formica subpolita*	Male-male copulation	Field	None	None	[129]
Arthropoda	Hymenoptera	*Habropoda laboriosa*	Male-male copulation	Field	None	None	[130]
Arthropoda	Hymenoptera	*Megarhyssa atrata*	Male-male copulation	Field	None	None	[131]
Arthropoda	Hymenoptera	*Megarhyssa macrurus*	Male-male copulation	Field	None	None	[131]
Arthropoda	Hymenoptera	*Osmia cornuta, O. bicornis*	Male-male copulation	Lab	Mistaken identity	None	[132]
Arthropoda	Hymenoptera	*Psyttalia concolor*	Male-male courtship	Lab	Adaptive	None	[29,112]
Arthropoda	Hymenoptera	*Psyttalia concolor*	Male-male courtship	Lab	Adaptive	None	[29,112]
Arthropoda	Hymenoptera	*Psyttalia concolor*	Male-male and female-female courtship	Lab	Adaptive	None	[112,133]
Arthropoda	Isoptera	*Coptotermes spp.*	Male-male and female-female tandem	Lab	Social and environmental influences (pheromones)	Positive	[134]
Arthropoda	Isoptera	*Not specified (termites)*	Male-male and female-female pairing	Lab	Adaptive	Positive	[135]
Arthropoda	Isoptera	*Reticulitermes chinensis*	Tandem running	Lab	Adaptive	Positive	[136]
Arthropoda	Isoptera	*Reticulitermes flavipes*	Male-male pairing	Lab	Adaptive	Positive	[12]
Arthropoda	Isoptera	*Reticulitermes speratus*	Tandem running	Lab	Adaptive	Positive	[137]

Arthropoda	Isoptera	*Reticulitermes speratus*	Female-female tandem	Lab	Social context	Variable and complex	[9]
Arthropoda	Lepidoptera	*Acraea andromacha*	Male-male courtship	Field	None	None	[138]
Arthropoda	Lepidoptera	*Acraea encedon*	Female-female copulation	Field	None	None	[139]
Arthropoda	Lepidoptera	*Acrolepiopsis assectella*	Male-male copulation	Field	None	None	[140]
Arthropoda	Lepidoptera	*Antheraea mylitta*	Male-male copulation	Field	None	None	[141]
Arthropoda	Lepidoptera	*Bicyclus anynana*	Male-male courtship	Field	None	None	[142]
Arthropoda	Lepidoptera	*Bombyx mori*	Male-male copulation	Field	None	None	[143]
Arthropoda	Lepidoptera	*Choristoneura fumiferana*	Male-male copulation and courtship	Field	None	None	[144]
Arthropoda	Lepidoptera	*Copitarsia decolora*	Male-male copulation	Field	None	None	[145]
Arthropoda	Lepidoptera	*Corcyra cephalonica*	Male-male copulation	Field	None	None	[146]
Arthropoda	Lepidoptera	*Cyaniris semiargus maroccana*	Male-male copulation	Field	None	None	[147]
Arthropoda	Lepidoptera	*Danaus erippus*	Male-male copulation	Field	None	None	[148]
Arthropoda	Lepidoptera	*Danaus gilippus berenice*	Male-male courtship	Field	None	None	[149]
Arthropoda	Lepidoptera	*Danaus plexippus*	Male-male copulation	Field	None	None	[148,150]
Arthropoda	Lepidoptera	*Eucheira socialis*	Male-male copulation	Field	None	None	[151]
Arthropoda	Lepidoptera	*Euphydryas aditha*	Male-male courtship and copulation	Field	None	None	[152]
Arthropoda	Lepidoptera	*Euphydryas anicia*	Male-male copulation	Field	None	None	[153]
Arthropoda	Lepidoptera	*Eupoecilia ambiguella*	Male-male courtship	Field	None	None	[154]

Arthropoda	Lepidoptera	*Grapholitha molesta*	Male-male copulation	Field	None	None	[155]
Arthropoda	Lepidoptera	*Heliconius charithonia*	Male-male copulation	Field	None	None	[156]
Arthropoda	Lepidoptera	*Jalmenus evagoras*	Male-male copulation	Field	None	None	[157]
Arthropoda	Lepidoptera	*Junonia coenia*	Male-male courtship	Field	None	None	[158]
Arthropoda	Lepidoptera	*Laspeyresia pomonella*	Male-male copulation	Field	None	None	[155]
Arthropoda	Lepidoptera	*Pieris brassicae*	Male-male copulation	Field	None	None	[159]
Arthropoda	Lepidoptera	*Pieris rapae crucivora*	Male-male copulation	Field	None	None	[1]
Arthropoda	Lepidoptera	*Pieris rapae rapae*	Male-male copulation	Field	None	None	[160]
Arthropoda	Lepidoptera	*Zeiraphera diniana*	Male-male copulation	Field	None	None	[155]
Arthropoda	Littorinimorpha	*Crepidula fornicata*	Male-male copulation	Lab	None	None	[161]
Platyhelminthes	Macrostomida	*Macrostomum hystrix*	Hypodermic self-insemination (related to SSB)	Field	Adaptive	NA	[19]
Arthropoda	Microcoryphia	*Promesomachilis hispanica*	Male-male copulation	Field	None	None	[162]
Acanthocephala	Moniliformida	*Moniliformis dubius*	Male-male copulation	Field	None	None	[26]
Arthropoda	Neuroptera	*Chrysopa carnea*	Male-male copulation	Field	None	None	[163]
Arthropoda	Octopoda	2 Unendentified Incirrata family	Male-male copulation	Field	None	None	[164]
Arthropoda	Odonata	*Calopteryx haemorrhoidalis*	Male-male copulation	Field	None	None	[165]
Arthropoda	Odonata	*Calopteryx splendens*	Male-male tandem	Field	Adaptive	None	[166]
Arthropoda	Odonata	*Cercion hieroglyphicum*	Male-male copulation	Field	None	None	[165]
Arthropoda	Odonata	*Ceriagrion nipponicum*	Male-male copulation	Field	None	None	[165]

Arthropoda	Odonata	*Ceriagrion tenellum*	Male-male copulation	Field	None	None	[165]
Arthropoda	Odonata	*Enallagma cyathigerum*	Male-male copulation	Field	None	None	[165]
Arthropoda	Odonata	*Gomphus adelphus*	Male-male copulation	Field	None	None	[165]
Arthropoda	Odonata	*Gomphus apomyius*	Male-male copulation	Field	None	None	[165]
Arthropoda	Odonata	*Gomphus dilatatus*	Male-male copulation	Field	None	None	[165]
Arthropoda	Odonata	*Gomphus geminatus*	Male-male copulation	Field	None	None	[165]
Arthropoda	Odonata	*Gomphus lineatifrons*	Male-male copulation	Field	None	None	[165]
Arthropoda	Odonata	*Gomphus modestus*	Male-male copulation	Field	None	None	[165]
Arthropoda	Odonata	*Gomphus ozarkensis*	Male-male copulation	Field	None	None	[165]
Arthropoda	Odonata	*Gomphus parvidens*	Male-male copulation	Field	None	None	[165]
Arthropoda	Odonata	*Gomphus vastus*	Male-male copulation	Field	None	None	[165]
Arthropoda	Odonata	*Gomphus viridifrons*	Male-male copulation	Field	None	None	[165]
Arthropoda	Odonata	*Hagenius brevistylus*	Male-male copulation	Field	None	None	[165]
Arthropoda	Odonata	*Ischnura elegans*	Male-male copulation	Lab	Social context	Positive	[167]
Arthropoda	Odonata	*Ischnura elegans*	Male-male copulation	Field	None	None	[165]
Arthropoda	Odonata	*Ischnura graellsii*	Male-male copulation	Field	None	None	[165]
Arthropoda	Odonata	*Ischnura senegalensis*	Male-male copulation	Field	None	None	[165]
Arthropoda	Odonata	*Lestes barbarus*	Male-male copulation	Field	None	None	[165]
Arthropoda	Odonata	*Lestes disjunctus*	Male-male copulation	Field	None	None	[165]
Arthropoda	Odonata	*Lestes sponsa*	Male-male copulation	Field	None	None	[165]

Arthropoda	Odonata	*Lestes viridis*	Male-male copulation	Field	None	None	[165]
Arthropoda	Odonata	*Leucorrhinia caudalis*	Male-male copulation	Field	None	None	[165]
Arthropoda	Odonata	*Leucorrhinia dubia*	Male-male copulation	Field	None	None	[165]
Arthropoda	Odonata	*Leucorrhinia hudsonica*	Male-male copulation	Field	None	None	[165]
Arthropoda	Odonata	*Leucorrhinia rubicunda*	Male-male copulation	Field	None	None	[165]
Arthropoda	Odonata	*Nehalennia gracilis*	Male-male copulation	Field	None	None	[165]
Arthropoda	Odonata	*Sympecma paedisca*	Male-male copulation	Field	None	None	[165]
Arthropoda	Odonata	*Sympetrum striolatum*	Male-male copulation	Field	None	None	[165]
Arthropoda	Odonata	*Trigomphus melampus*	Male-male copulation	Field	None	None	[165]
Arthropoda	Opiliones	Non identified	Male-male copulation	Field	None	None	[90]
Arthropoda	Orthoptera	*Acheta firmus*	Male-male copulation	Field	None	None	[168]
Arthropoda	Orthoptera	*Endecous chape*	Male-male courtship	Lab	Mistaken identity	None	[169]
Arthropoda	Orthoptera	*Gryllus veletis*	Male-male courtship and mounting	Lab	Mistaken identity	None	[170]
Arthropoda	Orthoptera	*Locusta migratoria*	Male-male copulation	Field	None	None	[171]
Arthropoda	Orthoptera	*Schistocerca gregaria (Desert locust)*	Male-male mounting	Lab	Social context	None	[10]
Arthropoda	Orthoptera	*Teleogryllus occipitalis (Crickets)*	Male–male courtship	Field and lab	Adaptive	None	[172]
Arthropoda	Orthoptera	*Teleogryllus oceanicus*	Male-male mounting and courtship	Lab	Indiscriminate mating	Negative	[6]
Arthropoda	Orthoptera	*Teleogryllus oceanicus*	Male-male copulation	Lab	Indiscriminate mating	None	[11]
Arthropoda	Orthoptera	*Teleogryllus oceanicus*	Male-male copulation	Lab	Indiscriminate mating	None	[14]

Arthropoda	Orthoptera	*Xenogryllus marmoratus*	Male-male mounting	Field	Social context	None	[173]
Acanthocephala	Polymorphida	*Polymorphus minutus*	Male-male copulation	Field	None	None	[26]
Acanthocephala	Rhabditida	*Nippostrongylus brasiliensis*	Female-female chemotatic attraction and male-male pairing	Field	None	None	[27]
Arthropoda	Rhabditida	*Caenorhabditis spp*	Male-male copulation	Field	Adaptive	None	[174]
Arthropoda	Rhabditida	*Cylindrocorpus longistoma* and *C. curzii*	Male-male copulation	Lab	Mistaken identity	None	[175]
Arthropoda	Siphonaptera	*Ceratophyllus gallinae*	Male-male copulation	Field	None	None	[176]
Arthropoda	Orthoptera	*Gryllus* spp (8 species)	Male-male courtship	Laboratory	Social context	None	[177]
Arthropoda	Orthoptera	*Teleogryllus oceanicus*	Female-female sexual behavior	Laboratory	Social context	None	[178]
Arthropoda	Diptera	*Drosophila santomea*	Male-male courtship	Laboratory	Adaptive	None	[179]

Table S2 List of 85 Orders with No Reported SSB used to explore phylogenetic signals in the evolutionary conservatism of SSB

Order	Common Name/Group	Phylum
Actiniaria	Sea anemones	Cnidaria
Alcyonacea	Soft corals	Cnidaria
Amphipoda	Scuds, sideswimmers	Arthropoda
Anostraca	Fairy shrimp	Arthropoda
Architaenioglossa	Land and freshwater snails	Mollusca
Arhynchobdellida	Jawless leeches	Annelida
Astigmatina	Mites	Arthropoda
Calanoida	Copepods	Arthropoda
Capitellida	Polychaete worms	Annelida
Cheilostomatida	Bryozoans (moss animals)	Bryozoa
Chitonida	Chitons	Mollusca
Cladocera	Water fleas	Arthropoda
Collembola	Springtails	Arthropoda
Comatulida	Feather stars	Echinodermata
Cumacea	Cumaceans	Arthropoda

Cyclopoida	Copepods	Arthropoda
Cyclophyllidea	Tapeworms	Platyhelminthe s
Cypridinida	Ostracods (seed shrimp)	Arthropoda
Diplopoda	Millipedes	Arthropoda
Diplostraca	Clam shrimp	Arthropoda
Dorylaimida	Predatory/omnivorous nematodes	Nematoda
Embioptera	Webspinners	Arthropoda
Enoplida	Free-living marine nematodes	Nematoda
Ephemeroptera	Mayflies	Arthropoda
Euphausiacea	Krill	Arthropoda
Forcipulatida	Sea stars	Echinodermata
Geophilomorpha	Soil centipedes	Arthropoda
Gorgonacea	Sea fans	Cnidaria
Haplosclerida	Demosponges	Porifera
Harpacticoida	Copepods	Arthropoda
Hirudinida	Leeches	Annelida
Holothuriida	Sea cucumbers	Echinodermata
Hydroida	Hydroids	Cnidaria
Isopoda	Woodlice, pill bugs	Arthropoda
Ixodida	Ticks	Arthropoda
Julida	Millipedes	Arthropoda
Limoida	File clams	Mollusca
Lithobiomorpha	Stone centipedes	Arthropoda
Lumbricida	Earthworms	Annelida
Lumbriculida	Freshwater worms	Annelida
Mantodea	Mantises	Arthropoda
Mecoptera	Scorpionflies	Arthropoda
Megaloptera	Dobsonflies, alderflies	Arthropoda

Mesogastropoda	Marine and freshwater snails	Mollusca
Mictacea	Crustaceans	Arthropoda
Mysida	Opossum shrimp	Arthropoda
Mytilida	Mussels	Mollusca
Nassellaria	Radiolarians	Retaria
Neogastropoda	Sea snails	Mollusca
Notostraca	Tadpole shrimp	Arthropoda
Nuculanida	Nut clams	Mollusca
Ophiurida	Brittle stars	Echinodermata
Orbiniida	Polychaete worms	Annelida
Ostreida	Oysters	Mollusca
Pectinida	Scallops	Mollusca
Pennatulacea	Sea pens	Cnidaria
Phasmida	Stick insects	Arthropoda
Phyllodocida	Polychaete worms	Annelida
Plecoptera	Stoneflies	Arthropoda
Poecilosclerida	Demosponges	Porifera
Polydesmida	Millipedes	Arthropoda
Protura	Coneheads	Arthropoda
Psocodea	Barklice, booklice	Arthropoda
Raphidioptera	Snakeflies	Arthropoda
Rhynchonellida	Brachiopods (lamp shells)	Brachiopoda
Sabellida	Feather duster worms	Annelida
Scleractinia	Stony corals	Cnidaria
Scolopendromorph a	Tropical centipedes	Arthropoda
Scorpiones	Scorpions	Arthropoda
Scutigeromorpha	House centipedes	Arthropoda
Solifugae	Camel spiders, wind scorpions	Arthropoda
Spatangoida	Heart urchins	Echinodermata

Spionida	Polychaete worms	Annelida
Spirobolida	Millipedes	Arthropoda
Stomatopoda	Mantis shrimp	Arthropoda
Strepsiptera	Twisted-wing parasites	Arthropoda
Stylommatophora	Land snails and slugs	Mollusca
Tanaidacea	Tanaids	Arthropoda
Terebellida	Spaghetti worms	Annelida
Thecostraca	Barnacles	Arthropoda
Thysanoptera	Thrips	Arthropoda
Tricladida	Planarians	Platyhelminthe s
Trichoptera	Caddisflies	Arthropoda
Zoraptera	Angel insects	Arthropoda
Zygentoma	Silverfish	Arthropoda

Table S3. PCA loadings for PC1 and PC2.

Variable	PC1	PC2	Interpretation
Phylum_Arthropoda	-0,082	0,936	Arthropod records
Study type_Field	0,881	-0,082	Field-based records
Study type_Laboratory	-0,834	0,081	Laboratory-based records
Behavior category_Copulation-like	0,807	0,276	Copulation-like reports
Phylum_Echinodermata	-0,03	-0,635	Echinoderm records
Behavior category_Attraction/aggregation	0,041	-0,62	Attraction/aggregation reports
Phylum_Acanthocephala	0,143	-0,617	Acanthocephalan records
Behavior category_Courtship	-0,603	0,092	Courtship reports
Behavior category_Mounting	-0,33	-0,175	Mounting reports
Behavior category_Pairing/tandem	-0,309	-0,29	Pairing/tandem reports
Phylum_Mollusca	0,035	-0,295	Mollusk records
Study type_Mixed	-0,235	0,012	Mixed field/laboratory records
Phylum_Annelida	-0,068	-0,154	Annelid records

Table S4. Software and computational packages used in the analytical workflow.

Software/package	Version	Analytical role	Reference(s)
R	4.3.2	Chi-square tests, odds ratios, logistic regression, phylogenetic signal analyses (as stated in Methods)	[180]
ape	to be verified in original R environment	Phylogenetic tree handling / comparative analyses	[181]
phytools	to be verified in original R environment	Phylogenetic comparative tools / Pagel's lambda	[182]
geiger	to be verified in original R environment	Blomberg's K / macroevolutionary comparative analyses	[183]
ggplot2	to be verified in original R environment	R figures / graphical summaries	[184]
dplyr	to be verified in original R environment	Data wrangling in R	[185]
tidyr	to be verified in original R environment	Data reshaping in R	[185]
Python	3.13.5	Re-created PCA/K-means environment and Supplementary Figure S3 generation in this container	[186]
pandas	2.2.3	Data handling in Python	[187]
numpy	2.3.5	Numerical operations in Python	[188]
scikit-learn	1.8.0	Principal Component Analysis and K-means clustering	[189]
matplotlib	3.10.8	Figure generation in Python	[190]
seaborn	0.13.2	Python visualizations	[191]

Figure S1. PRISMA flow diagram summarizing the identification, screening, eligibility assessment, and inclusion of studies in the systematic review. Records excluded during title and abstract screening (n = 394) corresponded to publications that did not meet the eligibility criteria for full-text assessment, including studies outside the taxonomic scope of the review, records not reporting same-sex sexual behavior or comparable sexual interactions in invertebrates, articles lacking original behavioral information, and publications not relevant to the objectives of the synthesis. Based on: [20]

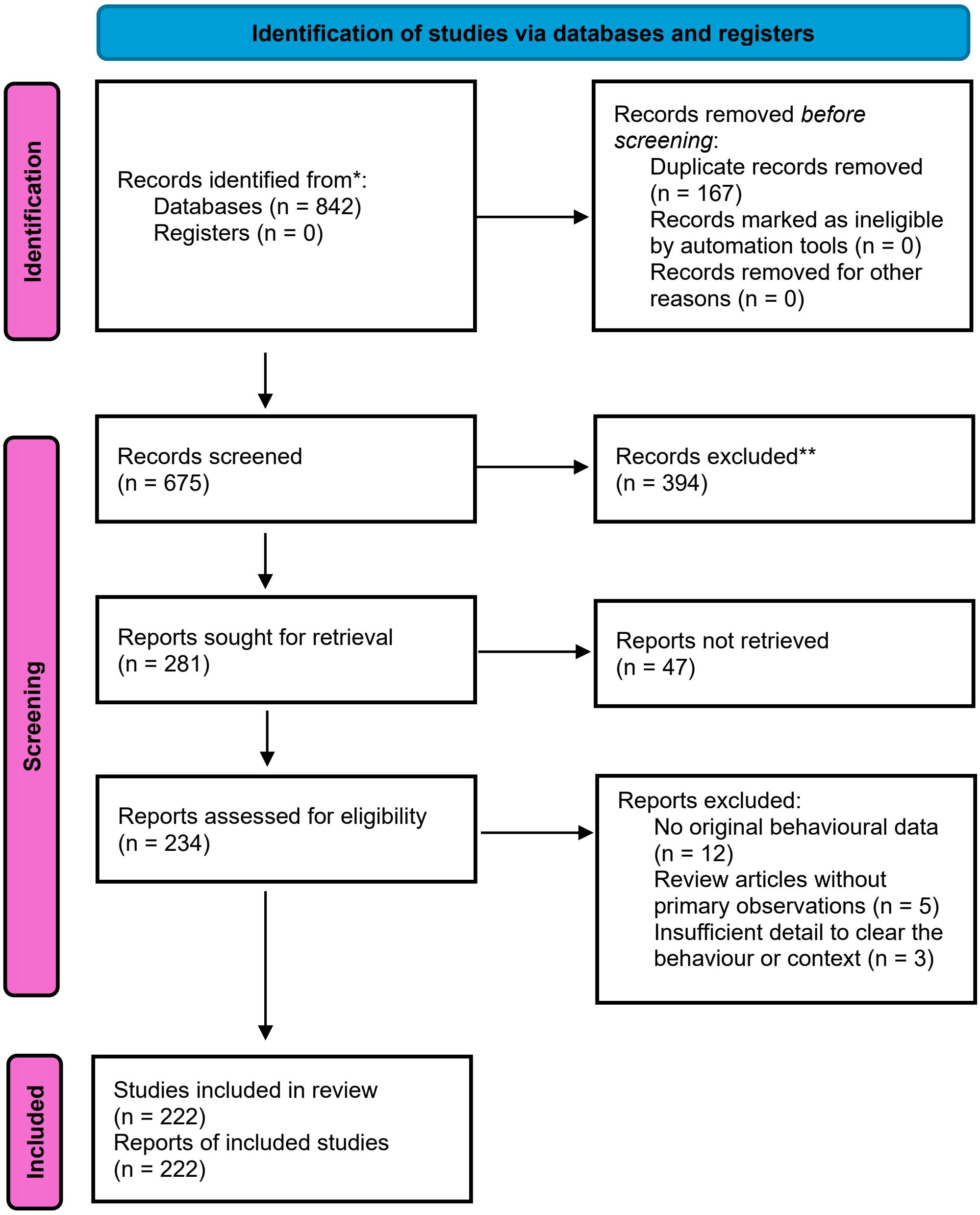

Figure S2. Relative Reporting Index (RRI × 1000) for SSB across 24 invertebrate orders. This visualization highlights both strong taxonomic biases in research effort and the uneven distribution of SSB documentation across invertebrate lineages.

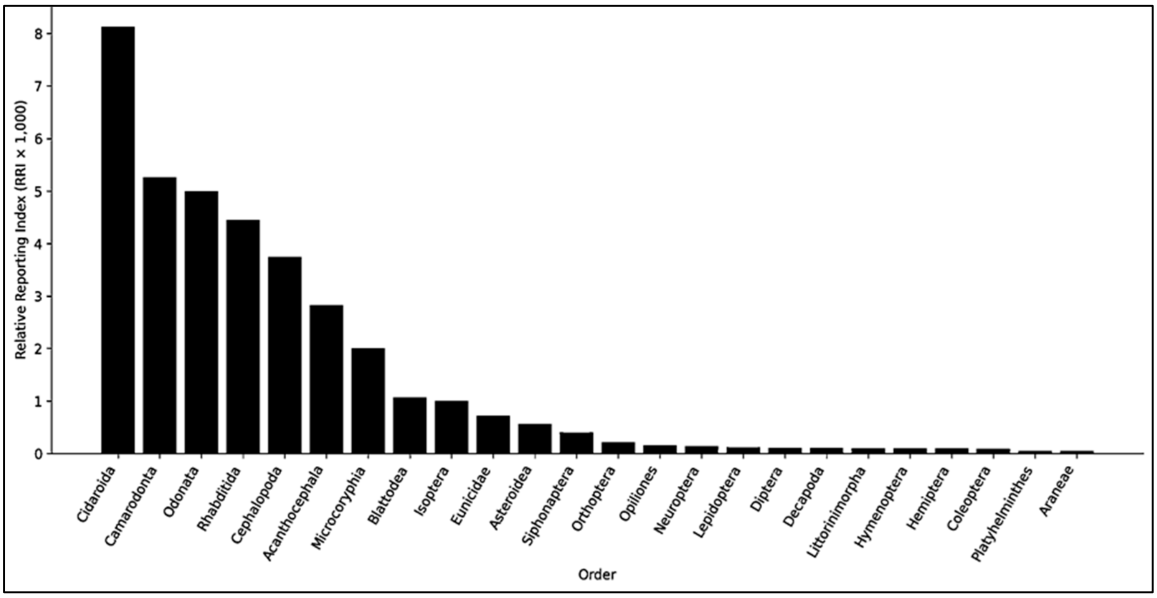

Figure S3. Synthetic order-level phylogenetic backbone used for exploratory phylogenetic signal analyses. The tree includes the 112 terminal orders or higher taxonomic groups used in the binary matrix: 27 with published SSB records and 85 representative orders or groups with no reports in the dataset. Filled circles indicate orders or groups with documented SSB records, whereas open circles indicate representative orders or groups without documented SSB records. Branch lengths were treated as equal and should not be interpreted as divergence-time estimates. The tree was used only to evaluate broad phylogenetic clustering in reporting patterns, not to infer evolutionary transitions or ancestral states.

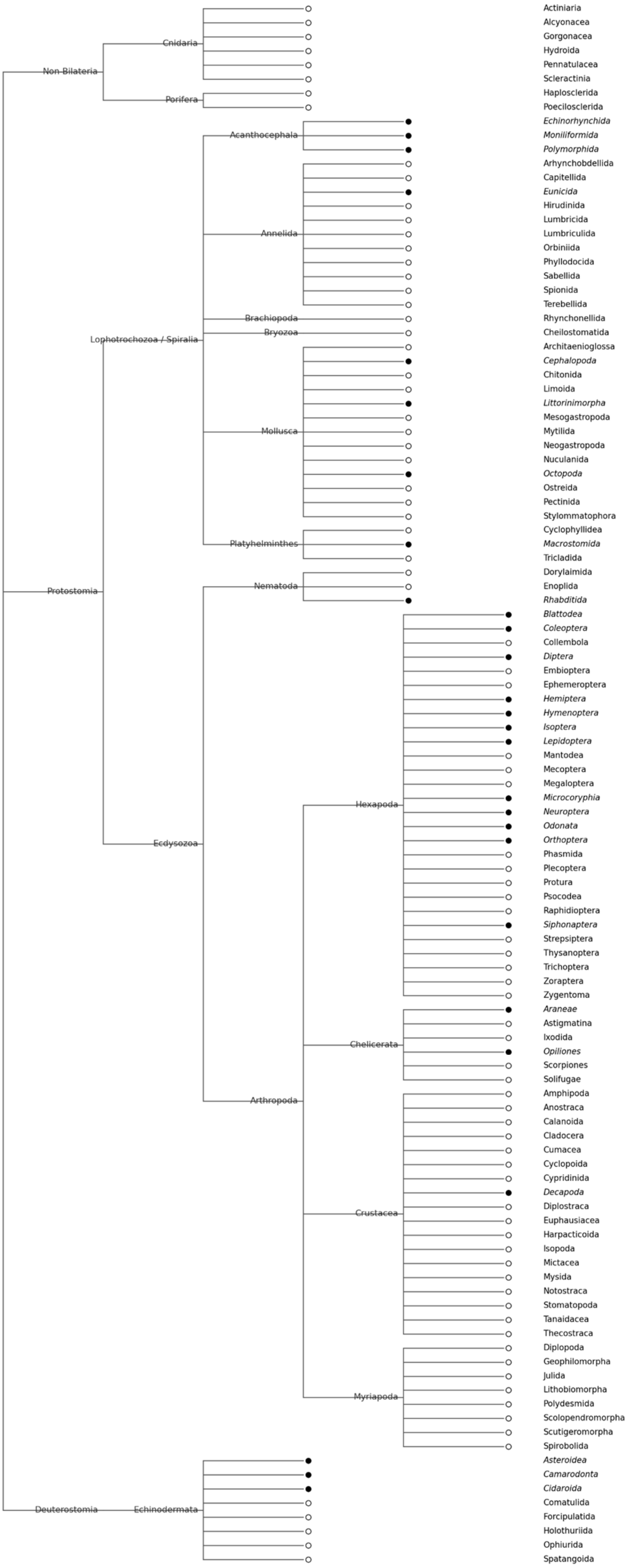
Non-Bilateria
Cnidaria
Porifera
Protostomia
Lophotrochozoa / Spiralia
Acanthocephala
Annelida
Brachiopoda
Bryozoa
Mollusca
Platyhelminthes
Ecdysozoa
Nematoda
Arthropoda
Hexapoda
Chelicerata
Crustacea
Myriapoda
Deuterostomia
Echinodermata
Actiniaria
Alcyonacea
Gorgonacea
Hydroida
Pennatulacea
Scleractinia
Haplosclerida
Poecilosclerida
Echinorhynchida
Moniliformida
Polymorphida
Arhynchobdellida
Capitellida
Eunicida
Hirudinida
Lumbricida
Lumbriculida
Orbiniida
Phyllodocida
Sabellida
Spionida
Terebellida
Rhynchonellida
Cheilostomatida
Architaenioglossa
Cephalopoda
Chitonida
Limoida
Littorinimorpha
Mesogastropoda
Mytilida
Neogastropoda
Nuculanida
Octopoda
Ostreida
Pectinida
Stylommatophora
Cyclophyllidea
Macrostomida
Tricladida
Dorylaimida
Enoplida
Rhabditida
Blattodea
Coleoptera
Collembola
Diptera
Embioptera
Ephemeroptera
Hemiptera
Hymenoptera
Isoptera
Lepidoptera
Mantodea
Mecoptera
Megaloptera
Microcoryphia
Neuroptera
Odonata
Orthoptera
Phasmida
Plecoptera
Protura
Psocodea
Raphidioptera
Siphonaptera
Strepsiptera
Thysanoptera
Trichoptera
Zoraptera
Zygentoma
Araneae
Astigmatina
Ixodida
Opiliones
Scorpiones
Solifugae
Amphipoda
Anostraca
Calanoida
Cladocera
Cumacea
Cyclopoida
Cypridinida
Decapoda
Diplostraca
Euphausiacea
Harpacticoida
Isopoda
Mictacea
Mysida
Notostraca
Stomatopoda
Tanaidacea
Thecostraca
Diplopoda
Geophilomorpha
Julida
Lithobiomorpha
Polydesmida
Scolopendromorpha
Scutigeromorpha
Spirobolida
Asteroidea
Camarodonta
Cidaroida
Comatulida
Forcipulatida
Holothuriida
Ophiurida
Spatangoida
